# Electrospun CNF Supported Ceramics as Electrochemical Catalysts for Water Splitting and Fuel Cell: A Review

**DOI:** 10.3390/polym12010238

**Published:** 2020-01-19

**Authors:** Sahil Verma, Sumit Sinha-Ray, Suman Sinha-Ray

**Affiliations:** 1 School of Engineering, Indian Institute of Technology Mandi, Mandi HP 175075, India; shlverma14@gmail.com; 2 Department of Mechanical and Industrial Engineering, University of Illinois at Chicago, Chicago, IL 60607, USA; 3 Corporate Innovation Center, United States Gypsum, Libertyville, IL 60048, USA

**Keywords:** electrospining, nanomaterials, carbon nanofibers, ceramic electrocatalyst, energy conversion

## Abstract

With the per capita growth of energy demand, there is a significant need for alternative and sustainable energy resources. Efficient electrochemical catalysis will play an important role in sustaining that need, and nanomaterials will play a crucial role, owing to their high surface area to volume ratio. Electrospun nanofiber is one of the most promising alternatives for producing such nanostructures. A section of key nano-electrocatalysts comprise of transition metals (TMs) and their derivatives, like oxides, sulfides, phosphides and carbides, etc., as well as their 1D composites with carbonaceous elements, like carbon nanotubes (CNTs) and carbon nanofiber (CNF), to utilize the fruits of TMs’ electronic structure, their inherent catalytic capability and the carbon counterparts’ stability, and electrical conductivity. In this work, we will discuss about such TM derivatives, mostly TM-based ceramics, grown on the CNF substrates via electrospinning. We will discuss about manufacturing methods, and their electrochemical catalysis performances in regards to energy conversion processes, dealing mostly with water splitting, the metal–air battery fuel cell, etc. This review will help to understand the recent evolution, challenges and future scopes related to electrospun transition metal derivative-based CNFs as electrocatalysts.

## 1. Introduction

Electrochemical catalysts, also known as electrocatalysts, are catalysts that actively participate in electrochemical reactions [1,2]. As the name suggests, the intent of the electrocatalyst is to participate and enhance the reaction rate without being consumed. In electrochemistry, the electrochemical catalysts are either a part of the electrode, or they are the electrode by itself. The process of electrochemical reactions can be dated back to the 1850s, where in 1860 the patent of the electrolysis of water [3] into hydrogen and oxygen was filed. However, in the last few decades, there has been a significant upsurge of research activity to develop efficient electrochemical catalysts, where the applications range spans between CO_2_ reduction [4], microbial fuel cells [5], proton exchange membrane fuel cells [6], water splitting [7], metal–air batteries [8] and many others. However, one of the biggest impetuses behind the sudden upsurge in the field of electrocatalysis can be attributed to the energy demand from renewable sources and the continuous push to move away from fossil fuels. According to the Global Energy Statistical Yearbook the total world demand for energy in 2018 was 14,046 Mtoe (“Mtoe” means million tons of oil equivalent) with a growth of 2.3% per year. A significant part of this world energy stems from a demand in electricity, where the energy demand grew by 4% [9].

It can also be seen that out of total energy demand, renewable sources amount for a meager 26% of generated electricity, while fossil fuels contribute a staggering amount of 64%. One of the key attributes of the dependence of energy derived from fossil fuel is not only the fact that it unsustainable, owing to the finite nature of stock, but also the environmental impact is significant. As a result of which, there has been an increasing demand of energy from nontraditional and renewable resources. Electrocatalysis is one of the means to achieve that goal line. Although electrocatalysis is an attractive option for renewable resources, traditional electrocatalysts suffer few challenges, namely, a smaller number of active sites, a corrosive environment [10] and their high temperature of operation [11].

In nanomaterials, as the surface area to volume ratio is significantly higher in comparison to that of a bulk material, it provides a significantly higher number of catalytically active sights for electrocatalysis. In that way, nanomaterials solve the challenge associated with a number of active sites. Additionally, nanomaterials also allow us to generate preferentially suitable materials for electrocatalysis. As a result of all of this, there has been a significant effort in developing suitable nanomaterials for electrocatalysis. Bottom-up chemical approaches like vapor liquid phase, hydrothermal and solvothermal techniques, which involve multiple steps, were exploited extensively in the recent past to synthesize a nanomaterial of desired shape and size [12], and the involvement of these additional synthesis steps affects the quality of nanomaterial formed, ultimately affecting the application efficiency they are going to be used for. Among the 0D, 1D, 2D nanomaterials, 1D nanomaterials, like nanofibers and nanowires, were found to show superior electrolyte diffusion and charge transportation when used for energy conversion and storage electrode material [13,14,15,16,17,18,19]. 1D-structured nanomaterials act as an expressway for charge transport, providing enhanced charge collection owing to their unique dimensionality [20]. Various routes like electrospinning, molecular beam epitaxy, chemical vapor deposition and electrodeposition, have been used in the past for the preparation of 1D nanomaterials having the desired structure and orientation [21,22,23,24,25,26,27,28,29,30]. Electrospining is one of the most promising methods for the synthesis of nanofibers, which can produce fibers of tunable length, diameter and composition; moreover, this versatile technique can be easily scaled up to give a high yield of nanofibers at a continues rate. Electrospining gives the edge over other methods by- (a) providing nanofibers in a persistent manner, (b) core shell geometry, (c) variable diameter, (d) the doping of nanofibers [31,32,33].

In order to alleviate the challenges associated with high temperature of operation and corrosive environment, TM oxides/sulfides/carbides, etc., are considered to be suitable alternatives to modern commercial electrocatalysts like platinum (Pt) or Pt-group metals [34]. Pt-group metals are cost-ineffective, and are still the modern-day fuel cells, and many electrochemical applications tend to bear the Pt-group metals as catalysts in their electrodes. However, their long-term durability, tending to agglomeration, CO poisoning, methanol intolerance, etc., render them inefficient for practical usage. Yet, their complete replacement is not exercised so far, despite the fact that thousands of research articles and patents have been registered under the notion of having a Pt-free electrocatalyst. Alternatively, TM oxides/sulfides/carbides, etc., have several advantages, like their tolerance to acidic or basic media, good electronic and protonic conductivity, and in terms of nanostructure, several synthesis techniques allow them to have high surface area [35]. TM phosphides and chalcogenides have even emerged as better electrical conductors, which render them to be more efficient than TM oxides [36] in terms of water splitting reactions. It is a fact that TM phosphides and chalcogenides may potentially convert into TM hydroxide during anodic oxidation, containing subsurface level TM phosphides and chalcogenides, facilitating electron transport at the interface, and making them even more interesting for further research [37]. 

However, all of the effects are intertwined with the surface area, porosity and distribution of such TM derivatives over another support/conductive host/conductor. Often such supporting networks are carbon derivatives, like CNTs, graphene etc. Amongst all of the carbon materials, CNFs have emerged as potential host/support materials for these TM derivatives due to their unique 1D structure, high electronic and thermal conductivities, and good electrochemical stability [38]. Especially, electrospun CNFs, generally derived after the carbonization of Polyacrylonitrile (PAN), are rich with pyridinc nitrogen, which aids in electrochemical catalysis as a heteroatom dopant. 

On top of that, degree of carbonization often dictates the graphitic nature of the CNFs, which again elucidates the electrochemical stability of it [38]. One of the key eases of using electrospinning to develop TM-derivative nanofibers is that of the simplicity and ability of tuning the compositions by varying the stoichiometric ratio of the precursor materials [39,40,41,42,43,44,45,46,47,48,49,50,51,52,53,54,55,56,57,58]. However, the challenge is that, owing to the brittle nature of such ceramic nanofiber produced via electrospinning, it is difficult to handle and process as a free-standing electrode. In this regard, a TM derivative-coated CNF offers an extremely attractive and practical solution to ease the handling issue by rendering mechanical and physicochemical stability, to provide the necessary high surface area for the TM derivative to distribute preventing their agglomeration, and often to enhance the electrical conductivity. 

In this review, we will discuss CNF-supported ceramic nanomaterials (TM derivatives) as electrocatalysts towards the Hydrogen Evolution Reaction (HER), Oxygen Evolution Reaction (OER), Hydrogen Oxidation Reaction (HOR) and Oxygen Reduction Reaction (ORR) for focused study on energy conversion, especially water splitting and fuel cell studies, as following. Initially, we will provide a brief background on electrochemical reactions pertaining to HER, OER, HOR and ORR for the brevity of this review. Following that, we will briefly describe the electrospinning process and finally present research efforts in the field of utilizing CNF-supported ceramic nanomaterial-based electrocatalysis involving the abovementioned domains of energy conversion, which will be discussed. At the end, we will conclude by describing the challenges and opportunities associated with this.

## 2. Brief Background of Electrocatalysis: HER, OER, HOR and ORR

An electrocatalyst may be defined as the substance, which when used as an electrode material in an electrochemical reaction, lowers the activation energy of the reaction [59]. The basic novelty in designing an electrocatalyst lies in developing unique architecture and active surface sites which play an important role in enhancing the electron transfers ability of the catalyst. The electrocatalyst can be of different forms, like that of a photoelectrocatalyst, a solution phase electrocatalyst, surface electrocatalyst, and many others [60,61,62,63,64]. Such electrocatalysts should be cheap, stable, selective and abundant enough to be used in large scale applications. For brevity, the review mostly outlines electrospun, CNF-supported, ceramic, nanoceramic materials for energy conversion, and hence, the bottom discussion will cover HER, OER and ORR mechanisms for mostly hydrolyzing (aka hydrogen generation) and fuel cells. 

### 2.1. Hydrogen Evolution Reaction and Oxygen Evolution Reaction

Hydrogen as an alternative fuel is lucrative for its nonpolluting and ecofriendly nature, which has a lot to offer in the domain of zero-carbon energy transport, especially in conjunction with fuel cells to meet future demands in the automobile, transport and heat industries, because of its high gravimetric energy density [65] The most simple and abundant method of the production of hydrogen is via the electrolysis of water. This electrolysis process requires a thermodynamic potential (E^0^) of 1.23 V at 25 °C and 1 atmospheric pressure, regardless of the choice of media, alkaline or acidic, and the total reaction is given below [66,67]:

H_2_O (l) → H_2_ (g) + ½ O_2_ (g) (1)

ΔG^0^ = +237.2 kJ mol^−1^, E^0^ = 1.23 V.

The process of electrolysis is initiated via two reactions which include the hydrogen evolution reaction (HER) and the oxygen evolution reaction (OER). In acidic medium, the water splitting reactions can be described as,

(2) $$\begin{array}{l} 2H^{+}\left(\mathrm{aq}\right)+2e^{-}\to H_{2}\left(g\right)\;(\mathrm{Cathodic})\\ 2H_{2}O\left(l\right)\to 4e^{-}+4H^{+}\left(aq\right)+\;O_{2}\left(g\right)\;(\mathrm{Anodic}) \end{array}\}$$

In basic or neutral medium, the same reactions can be described as,

(3) $$\begin{array}{l} 2H_{2}O\left(l\right)+4e^{-}\to H_{2}\left(g\right){+2\mathrm{OH}}^{-}\;(\mathrm{Cathodic})\\ 4OH^{-}\left(aq\right)\to 4e^{-}+4H^{+}\left(aq\right)+\;O_{2}\left(g\right)\;(\mathrm{Anodic}) \end{array}\}$$

The process of HER follows three possible reaction mechanisms in an acidic environment, albeit the same cannot be concluded for the alkaline medium [68,69]. In the former medium, the initialization of HER takes place with the Volmer step at the cathode where the electron combines with the proton, leading to an intermediate stage of hydrogen adsorption (H_ads_) on the catalyst surface. The Volmer step can then be forwarded via two possible pathways to produce H_2_–. (a) When the H_ads_ coverage is low on the surface, it will link with the electron and proton, leading to the formation of H_2_, namely following the Heyrovsky step, or (b) when the available H_ads_ coverage is high, they link up with the adjacent hydrogen atom and this produces H_2_, namely following the Tafel step [68]. For any pathway the Volmer path is necessary.

(4) $$\begin{array}{l} H^{+}+e^{-}\to H_{\mathrm{ads}}\left(\mathrm{Volmer}\;\mathrm{Step}\right)\\ H^{+}+H_{\mathrm{ads}}+e^{-}\to H_{2}\left(\mathrm{Heyrovsky}\;\mathrm{Step}\right)\\ 2H_{\mathrm{ads}}\to H_{2}\left(\mathrm{Tafel}\;\mathrm{Step}\right) \end{array}\}$$

**Figure 1:**
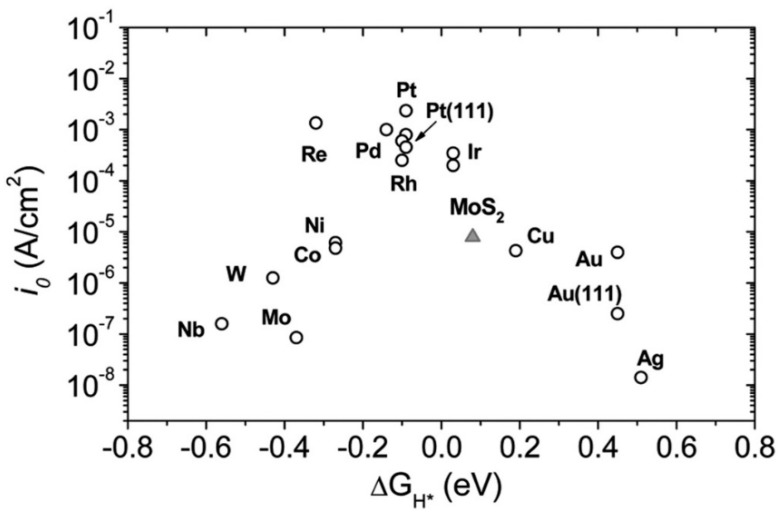
Various hydrogen evolution reaction (HER)-active materials with their exchange current density (*i*_0_) vs. hydrogen adsorption free energy (Δ*G*_H*_). Platinum (Pt) and Pt-group materials shows the highest value for HER activity among various materials. Reproduced with permission from Ref. [69]. Copyright Science 2007.

For HER, a certain overpotential than the thermodynamic requirement is necessary, depending on the catalyst’s activation barrier and other factors like solution resistance, etc. The overpotential arising from the polarization of the active electrode requires energy for electrolysis, which is the key parameter for deciding an electrocatalyst. The lower the value of the overpotential, the better is the catalyst, which can further largely tweaked by an available catalyst surface, for which 1D nanomaterials are often preferred [70]. The best known HER catalysts are Pt and Pt-group elements having nearly zero overpotential, as can be seen from Figure 1, and as mentioned in Strmcnik et al. (2016) [71]. But they are expensive and low in abundance. Hence, several researchers are trying to develop 1D and 2D nanomaterials, especially ones that are abundantly metal oxide-based, which have the optimum combination of surface area with near zero Δ*G*_H*_. 

In the OER process, H_2_O is oxidized to O_2_ following Equations (2) and (3), and irrespective of the medium, the reaction involves four consecutive proton coupling and electron transfer steps followed by oxygen bond formation, resulting in slow reaction kinetics [72,73]. As the theory suggests, the most common reaction steps involving Pt and oxygen reduction can be shown as following [72]:

(5) $$\begin{array}{l} O_{2}+H^{+}+e^{-}⇄OOH_{\mathrm{ads}}\\ OOH_{\mathrm{ads}}⇄OH_{\mathrm{ads}}+O_{\mathrm{ads}}\\ O_{\mathrm{ads}}+H^{+}+e^{-}⇄OH_{\mathrm{ads}}\\ OH_{\mathrm{ads}}+H^{+}+e^{-}⇄H_{2}O \end{array}\}$$

**Figure 2:**
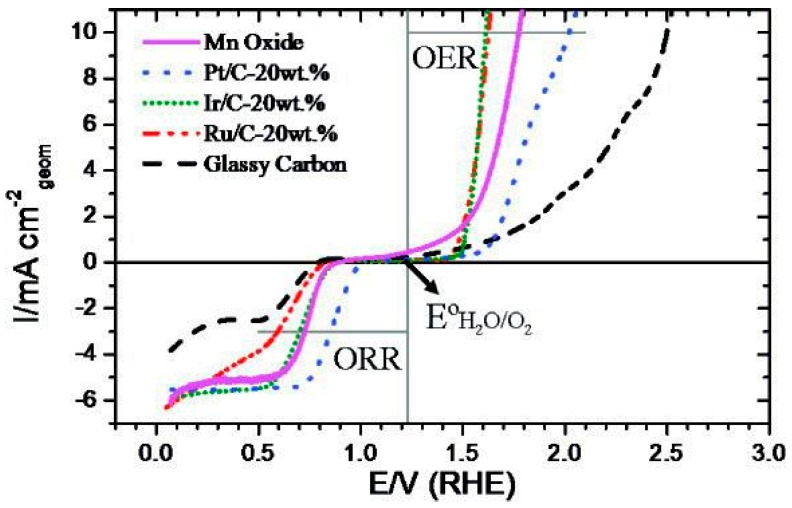
Oxygen electrode activities of the nanostructured Mn oxide thin film, nanoparticles of Pt, Ir, and Ru, and the GC substrate. The Mn oxide thin film shows excellent activity for both the oxygen reduction reaction (ORR) and the oxygen evolution reaction (OER). Reproduced with permission from Ref. [75]. Copyright, Journal of the American Chemical Society 2010.

Because of the energy difference of the above-mentioned intermediates OH and OOH of about 3.2 ± 0.2 eV, arising due to their non-interaction with the catalyst surface, an intrinsic overpotential of about 0.25–0.35 V exists for OER (Figure 2). Iridium- and ruthenium-based oxides are the leading benchmarks in an acidic medium for the OER, but they suffer from lower ORR activity [74].

### 2.2. Hydrogen Oxidation Reaction and Oxygen Reduction Reaction

As mentioned in the previous subsection, fuel cells have already renewed widespread attention, especially in the modern days’ automobile sectors like fuel cell electric vehicles (FCEV). The Hydrogen Oxidation Reaction (HOR) and Oxygen Reduction Reaction (ORR) are the key processes that take place on the anode and cathode of the most widely used proton exchange membrane fuel cell (PEMFC). For brevity, this review will concentrate on aqueous, electrolyte-based fuel cell studies, and not necessarily on other fuel cells, like direct methanol fuel cell (DMFC) etc., albeit they are popular as well [76]. 

In alkaline medium, HOR proceeds via three possible routes: (a) dissociative adsorption of H_2_ (Tafel step), (b) electron transfer from H_2_ to the catalyst (Heyrovsky step) and then (c) discharge of the adsorbed hydrogen atom (Volmer step). The processes can be described via the following reaction mechanisms [77]:

(6) $$\begin{array}{l} (a)\;H_{2}+2*\to 2\left(*-H_{\mathrm{ads}}\right)\\ (b)\;H_{2}+OH^{-}+2*\to \left(*-H_{\mathrm{ads}}\right)+H_{2}O\\ \;\;\;\;\;\;\mathrm{or}\\ \;\;H_{2}+\left(*-OH_{\mathrm{ads}}\right)⇄\left(*-H_{\mathrm{ads}}\right)+H_{2}O\\ \mathrm{followed}\;\mathrm{by}\\ \;\;OH^{-}+*⇄\left(*-OH_{\mathrm{ads}}\right)+e^{-}\\ (c)\;\left(*-H_{\mathrm{ads}}\right)+OH^{-}\to *+H_{2}O+e^{-} \end{array}\}$$

In Equation (6), the symbol * represents the catalytic surface site. Figure 1 is equally applicable for the HOR processes. Similarity in theoretically predicted exchange, current density values for the HOR and HER versus hydrogen adsorption free energy does not necessarily imply the similarity in the kinetics and mechanism of both of the reactions. However, apart from Pt group metals, Ni, a transition metal, and its oxides are deemed as potential reactive catalysts for HER/HOR [77].

ORR is a multiple-step process in which the presence of acidic or basic electrolyte will decide whether the product will be H_2_O or OH**^−^** [57,58]. In an acidic medium the O_2_ may directly convert to H_2_O via a 4-electron path or may undergo two steps involving the 2-electron path which leads to the formation of hydrogen peroxide (H_2_O_2_). Similarly, in an alkaline medium there may be a direct conversion to hydroxide (OH**^−^**) by the 4-electron path, or the reaction will follow a multistep path including the H_2_O**^-^** formation having 2-electron transfer in each step [78].

In an acidic medium:

(7) $$\begin{array}{l} (a)\;O_{2}+4H^{+}+4e^{-}\to 2H_{2}O\\ \;\;\;\;\;\mathrm{or}\\ (b)\;O_{2}+2H^{+}+2e^{-}\to H_{2}O_{2}\\ \;\;H_{2}O_{2}+2H^{+}+2e^{-}\to 2H_{2}O \end{array}\}$$

In an alkaline medium:

(8) $$\begin{array}{l} (a)\;O_{2}+2H_{2}O+4e^{-}\to 4OH^{-}\\ \;\;\;\;\;\;\mathrm{or}\\ (b)\;O_{2}+H_{2}O+2e^{-}\to HO_{2}{}^{-}+\;OH^{-\;}\\ \;\;HO_{2}{}^{-}+H_{2}O+2e^{-}\to 3OH^{-} \end{array}\}$$

Catalyzing with the 4-electron reduction path is an indicator of better electrocatalytic ORR efficiency, since the by-product of this mechanism is mostly preferred (water). In the conventional fuel cells, Pt/C (platinum as catalyst supported on conductive carbon) is chosen as the ORR catalyst because of its superior performance and its preferred 4-electron reduction path, despite the major cost of Pt and CO poisoning of the Pt/C-based electrode [78,79]. 

**Figure 3:**
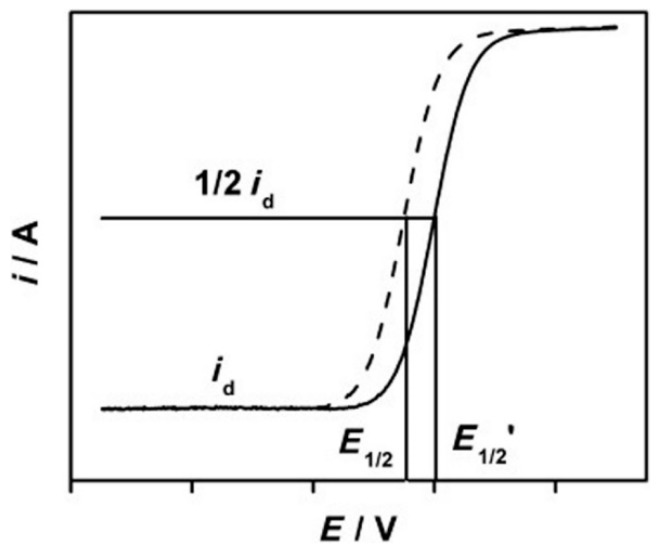
Polarization curve of two different catalysts in the oxygen reduction reaction (ORR) and the essential parameters which are needed for qualitative and quantitative analysis of their performances. Reproduced with permission from Ref. [80]. Copyright Angewandte Chemie International Edition 2013.

In order to elucidate the performance of catalysts in ORR, half-wave potential (E_1/2_) is generally obtained from the I-V polarization curve to qualitatively compare catalysts, where higher potential means better activity, as shown in Figure 3 [80]. In an alkaline solution, the rate of the time-dependent current drop measured at E_1/2_ is usually used to estimate the durability of the catalysts. For a better quantitative comparison between a standard catalyst, like Pt/C and a novel catalyst, the Koutecky–Levich (K–L) equation is used identify the kinetic current, from a polarization curve using a rotating disk electrode (RDE):

(9) $$\begin{array}{l} \frac{1}{J}=\frac{1}{J_{K}}+\frac{1}{J_{L}}=\frac{1}{J_{K}}+\frac{1}{B\sqrt{\omega }}\\ B=0.62nFD_{O_{2}}{}^{2/3}\nu^{-1/6}C_{0}\\ J_{K}=nFKC_{O} \end{array}\}$$

where *J* is the measured current density, and *J*_k_ and *J*_L_ represent the kinetic and diffusion-limiting current densities, respectively. In Equation (9), *n* stands for the electron transfer number (which indicates the reaction mechanism), *C*_0_ is the saturated concentration of oxygen in the medium, and F, ω, $D_{O_{2}}$, υ and K are the Faraday constant, which represents the rotating rate (rad/s), the diffusion coefficient of oxygen (1.9 × 10^−5^ cm^2^ s^−1^), the kinematic viscosity of the solution, and the rate constant for oxygen reduction, respectively.

## 3. Brief Background of Electrospinning 

**Figure 4:**
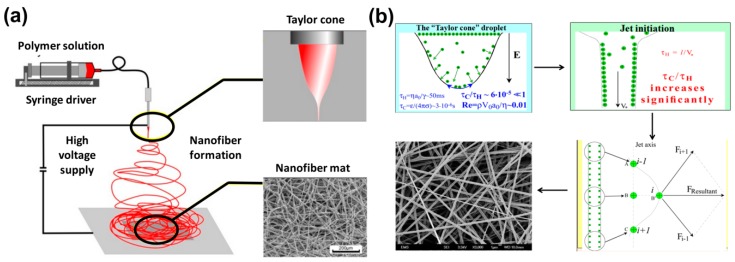
Schematic of (**a**) electrospinning and (**b**) the interaction of polymer and applied electric field is shown. Reproduced with permission from Ref. [84]. Copyright Springer Nature 2018.

Electrospinning is a unique method to produce polymer nanofibers from a wide variety of polymer solutions. Although electrospinning is mostly limited to polymer solutions at room temperature, there are few examples, where polymer nanofibers are prepared from polymer melts at elevated temperature [81], in liquid CO_2_ at low temperature [82,83,84], and other extreme conditions. However, irrespective of the methodologies, the basic mechanism of electrospinning remains the same. A detailed description can be obtained from refs.83 and 84. However, for the sake of brevity, it can be described as follows (Figure 4). In the case of electrospinning, polymer solutions are supplied through a nozzle (Figure 4a), which is connected to high voltage supply with an electric field ~1–2KV/ cm, and the distance between the polymer nozzle and the grounded collector plate is ~10–5 cm. Near the nozzle, the characteristic time of charge relaxation (τ_c_) << hydrodynamic time of residence (τ_H_), where τ_c_/τ_H_ ∼10^−5^ for a typical electrospinning process (Figure 4b). As a result of which, the polymer solution behaves as a perfect conductor. This zone is called the “Taylor Cone” [85]. However, after the Taylor Cone, in the polymer jet section, τ_c_/τ_H_ increases significantly. As a result of which, the polymer jet behaves as perfect dielectric material, where the charge resides underneath the free surface. During the process, any minute perturbation results in continued coulombic repulsion, as shown in Figure 4b. Subsequently, the polymer jet keeps on moving and stretching until the viscoelastic force pulls the polymer jet back. After that, owing to the similar mechanism, the stretching continues in the opposite direction. This occasions vigorous bending and flapping, as shown in Figure 4a, and results in polymer nanofibers (diameter ~100–500 nm) from an mm-sized polymer jet, as shown in Figure 4a,b. It needs to be mentioned that apart from the nozzle, the polymer jets can be produced without the nozzle also. One such example is described in [86], where the capillary breakup of polymer film over a rotating cylinder was used to create the polymer jet.

## 4. CNF-Supported Metal Oxides/Carbides/Phosphides/Sulfides as Electrocatalysts 

A variety of carbon materials with 1D architecture are being researched as efficient electrode materials for energy devices, because they provide a large surface to volume ratio, necessary electrical conductivity and electrochemical stability. Moreover, they can be utilized as conductive additives or substrates for supporting metal/metal-oxides and other ceramics [87,88,89]. On the other hand, as mentioned in Section 1, tunable properties of transition metal oxides, carbides, phosphides or sulfides in their nanoarchitecture offer high surface area, stability, increased activity, high porosity, ease of functionalization, etc., which make them suitable candidates for Pt/RuO_2_/IrO_2_-free catalyst synthesis for HER, OER, HOR and ORR studies. Among various other 1D carbon nanomaterials, electrospun carbon nanofibers (CNFs) are promising support materials for these metal oxides/nitrides, because of its ease of synthesis and heavily tortuous path that allows the electrolyte to cover a large surface of the fabric. It has been mentioned elsewhere that because of 1D alignment and the inherent porosity in the electrospun fibers, ionic transport gets faster and renders the catalyst, like even post-transition metal oxide, performing at a par with conventional Pt/C [19]. 

### 4.1. CNF Supported Metal Oxides 

**Figure 5:**
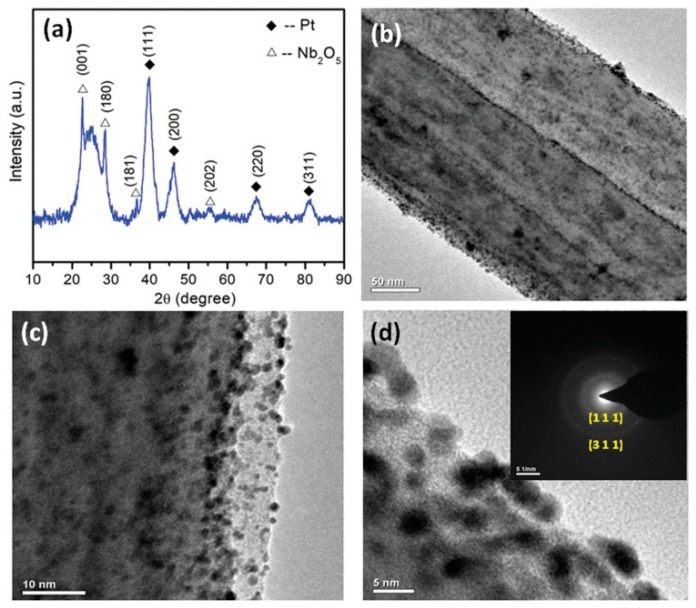
(**a**) X-Ray Diffractrogram (XRD) of Nb-CNF-Pt, (**b**–**d**) Transmission Electron Microscopy (TEM) images of Nb-CNF-Pt with inset of (**d**) representing the selected area electron diffraction (SAED) pattern of Nb-CNF-Pt. Reproduced with permission from Ref. [90]. Copyright, John Wiley and Sons 2019.

Shanmugapriya et al., 2019 [90], have demonstrated the catalytic activity of electrospun Nb_2_O_5_-incorporated CNFs as a support material for platinum nanoparticles towards HER, OER and ORR. The Nb-CNF-Pt was reported to outperform the commercial Pt/C-loaded carbon with a high positive onset potential of 0.99 V vs. a reversible hydrogen electrode (RHE) and E_1/2_ of 0.87 V vs. RHE during ORR. The multifunctional catalyst also demonstrated a minimal overpotential of 37 and 325 mV for HER and OER. The TEM images show that the Pt atoms have occupied the surface defects sites without being agglomerated, and are at present mostly at the edges of the fiber showing the high functionalization of CNF, as seen from Figure 5. The authors have claimed that the multi-functionality of the catalyst hails from the 1D nanostructure of CNF with nitrogen moiety favoring the electronic and electrochemical stability, coupled with the benefits of Nb_2_O_5_ co-participating in the catalytic activity, controlling the Pt nanoparticle dissolution, tuning the Pt electronic structure, and in conjunction with CNF, also increasing the corrosion resistance.

Jung et al. (2012) [91] had demonstrated the fabrication of elctrospun Mn_3_O_4_/C nanofibers in a one-step thermal treatment. The morphology, as revealed by Scanning Electron Microscopy, resembles shape of small, broken, yet smooth, fibers in the range of 200–300 nm. For the electrochemical tests, the author used Ketjen Black (KB) as a conducting agent. Mn_3_O_4_/C nanofibers were found to be active for the OER and ORR in 0.1 M KOH. Mn_3_O_4_/C nanofibers show an onset potential of 0.9 V in the case of the ORR, which is at par with a commercial Pt electrode. In the case of the OER, neither Mn_3_O_4_ + KB, CNF + KB participates significantly, where as, Mn_3_O_4_/C nanofibers demonstrate significant improvement because of enhanced catalytic surface. 

**Figure 6:**
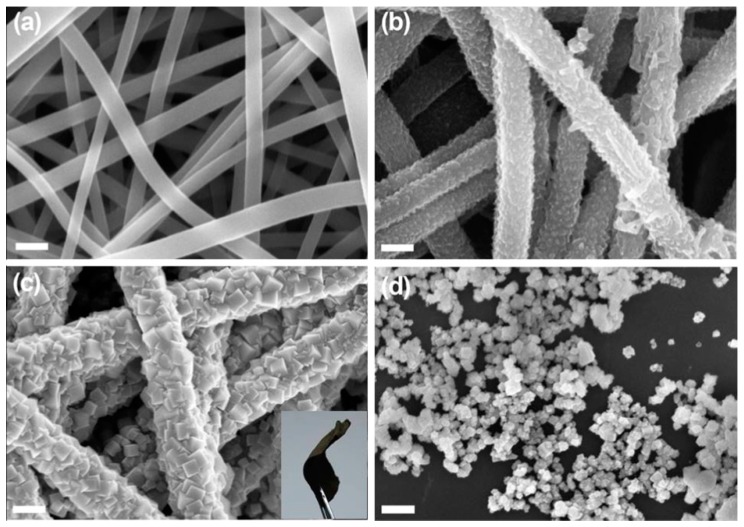
Field Emission Scanning Electron Microscope (FESEM) images of (**a**) electrospun carbon nanofibers, (**b**) NF-0.02, (**c**) NCNF-0.02@Co_3_O_4_-0.2 fiber membranes, and (**d**) Co_3_O_4_ powder. Inset (c) shows a free-standing NCNF-0.02@Co_3_O_4_-0.2 fiber membrane. Reproduced with permission from Ref. [93]. Copyright Elsevier 2016.

Apart from Mn-oxide, another transition metal oxide has been considerably investigated, and that is Co-oxide. Cobalt-based oxides are relatively known for their reactivity, and sufficient attention has been given over these particular materials as catalysts for ORR activities [92]. However, their poor conductive nature often causes their agglomeration, leading to sluggish kinetics. Miao et al. (2016) [93] prepared nitrogen-doped (polyaniline-coated fibers) CNF, coupled with Co_3_O_4_ nanoparticles denoted as NCNF@Co_3_O_4_, with the help of electrospinning followed by hydrothermal and heat treatment (Figure 6). The presence of the CNF yielded a free-standing network, leading to a binder free electrode and a less resistant ORR kinetic. Although the onset potential was reported to be −0.19 V vs. Ag/AgCl, the experimentation procedure could have its effect upon the final outcome, as the membrane was directly glued onto the glassy carbon as an active electrode, resulting in an inherent resistance. However, the electron transfer number (n) being 3.99 indicated towards an ideal 4-electron ORR pathway and highly superior performance in chronoamperometry, compared to commercial Pt/C, which elucidates the efficacy of the nitrogen-doped, CNF-supported Co_3_O_4_ network, where CNF provided the necessary electrical conductivity and acted as a backbone and prohibited Co_3_O_4_ dissolution or agglomeration. 

As mentioned previously, oxygen evolution is an important reaction for many practical applications like electrolysis or metal-air batteries. Since OER is sluggish by nature, and it occurs at the electrode–electrolyte interface, the large surface area and good conductivity of the catalyst are the major important criteria for OER [94]. Among different metal oxides, a significant amount of investigation has been conducted on mixed metal oxide spinels (A^2+^B^3+^_2_O_4_) supported on CNFS. CoFe_2_O_4_ nanoparticle-embedded N-doped carbon nanofibers (aka CoFe_2_O_4_@N-CNFs) were shown to be an efficient catalyst for OER [95]. Contrary to many researchers, Li et al. (2017) [95], had demonstrated the electrospinning technique using Polyvinylpyrrolidone (PVP), instead of Polyacrylonitrile (PAN). The Tafel slope of the CoFe_2_O_4_@N-CNFs was calculated to be 80 mV/dec, compared to that of commercial RuO_2_-75 mV/dec using 0.1 M KOH as the electrolyte. More importantly, the overpotential value at 10 mA/cm^2^ (analogus to a 10% efficient solar-to-fuel conversion device) were near identical; 349 mV for former vs. 342 for RuO_2_. However, at a greater current density, the said catalyst outperforms the commercial catalyst by a large margin. The superior performance of this particular non-precious metal oxide/CNF composite catalyst was attributed to a uniform dispersion of catalytically active spinel oxide and nitrogen-doped CNF that enhnaces the conductive and catalytic nature of the catalyst; especially the 1D architecture leading to the 3D network allowed more mass diffusion and electron transport. Another kind of spinel oxide containing nickel in the A site is also favorable, because of the low energy of Ni–O bond formation while interacting with –OH_ads_ during the OER. 

Busacca et al. (2019) [96] investigated the performance of two spine oxides NiMn_2_O_4_ and NiCo_2_O_4_ supported on carbon nanofibers in the OER. At 6 M KOH, the authors have reported as high as 62 and 25 A/g mass activity in oxygen evolution at 1.5 V for the former and latter catalysts, respectively. Like previously mentioned cases, CNF supported the morphology during the reaction and provided the conductive network. In a similar kind of study of OER improvement using a noble metal free catalyst, Wang, et al. (2015) [97] demonstrated the application of CoO/CNF as an efficient OER active catalyst. The least onset potential of 1.51 V was achieved using this system, which was mostly achieved because of the increased surface area, however within few galvanostatic cycles; the effect was reduced mostly because of loose CoO nanoparticles. 

**Figure 7:**
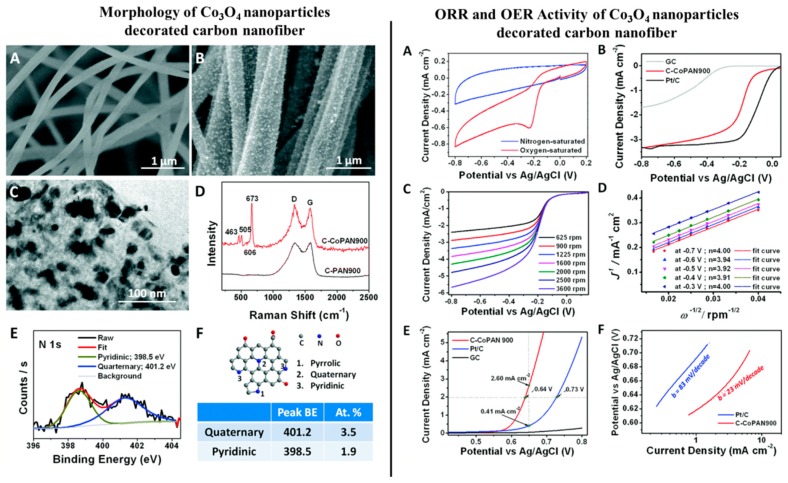
**Left** hand side: (**A**) Scanning electron microscopy (SEM) image of pure carbon nanofiber (CNF). (**B**) SEM image of Co (II)-decorated CNF. (**C**) Transmission electron microscopy (TEM) image of a single nanofiber of Co (II)-decorated CNF membrane. (**D**) Raman spectra of set (**A**) denoted by black line and set (**B**) denoted by red line with four of the characteristic peaks of crystalline Co_3_O_4_ visible in the latter. (**E**) High resolution X-ray photoelectron spectroscopy (XPS) spectrum of N1s for set (**A**). (**F**) Schematic illustration of the different N atoms doped in the carbon matrix. The table indicates the peak positions as well as the atomic % of quaternary and pyridinic nitrogen detected in set (**B**). In the **Right** hand side: Electrocatalytic performances of the catalyst Co (II)-decorated CNF. (**A**) Cyclic Voltammetry (CV) curves of Co (II)-decorated CNF in O_2_-saturated, (denoted by red line) and in N_2_-saturated (denoted by blue line) 0.1 M KOH solution. (**B**) Linear sweep voltammetry curves of the catalyst as compared to GC and commercial Pt/C for the ORR at electrode rotating speed of 900 rpm. (**C**) Rotating disk electrode (RDE) curves of the catalyst at various rotating speeds. (**D**) Koutecky–Levich plots derived from the RDE curves obtained in (**C**), following Equation (9). (**E**) Linear sweep voltammetry (LSV) curves of catalyst as compared to GC and commercial Pt/C for the OER at electrode rotating speed of 900 rpm. (**F**) Tafel slopes of C–CoPAN900 and Pt/C derived from (E). Reproduced with permission from Ref. [99]. Copyright, Royal Society of Chemistry 2017.

Li et al. (2015) [98] investigated the electrochemical performance of Co_3_O_4_-coated CNFs, where the metal–ion-containing polymer fibers were fabricated via the electrospinning technique followed by thermal carbonization and a post annealing process in air. The catalyst showed superior activity in both the OER and ORR due to: (a) Total quaternary and pyridinic nitrogen content > 5%, contributed by CNF leading to superior ORR activity and favoring 4-electron path and (b) uniform distribution of Co_3_O_4_ nanoparticles (5–50 nm) on CNFs, helping OER activity. The synergistic effect was clearly visible, as the catalyst exhibited E_1/2_ only 96 mV more negative than Pt/C (Vs Ag/AgCl) for ORR, and 90 mV more positive than the same catalyst at 2 mA/cm^2^ for OER [cf. Figure 7]. In 2017, the same group of authors showed the efficacy of Ni/NiO*_x_*-CNF architecture towards the OER and Zn–air batteries. [99]. In this work, the authors electrospun a polyacrylonitrile-bearing nickel precursor to fabricate the metal–ion-containing fibers, which upon carbonization at 900 °C in inert atmosphere and subsequent thermal treatment at 300 °C in air, revealed a NiO*_x_*-decorated CNF architecture. The said catalyst exhibited OER performance at a par with a commercial Ir-oxide/C catalyst, as at the current density of 10 mA/cm^2^, the former required 432 mV of overpotential, whereas the latter needed 398 mV. In a separate study, polyacrylonitrile (PAN)-based carbon (C)@NiO/Ni nanofibers were fabricated via the electrospinning method followed by thermal treatment for the HER purpose by Chinnappan et al. (2018) [100]. The catalyst reportedly performed better than pure Ni/NiO in terms of its Tafel slope, as the former reached 152 mV/dec, whereas for the latter the Tafel slope was reported to be 244 mV/dec elsewhere [101].

Tungsten dioxide (WO_2_) or trioxide (WO_3_) or suboxides (WO_3-*x*_) are known to have high metallic conductivity, and these are among the lucrative options in transition metal oxide groups [102]. However, they are prone to transform into tungsten carbide in high carbon concentration, and hence it is very difficult to synthesize them with CNFs, while carbonizing and yet retaining the suboxide structure [103]. Chen et al. (2016) [104] had demonstrated a novel technique to incorporate WO_3-*x*_ in a controlled manner on the electrospun CNFs, where with the increment of tungsten precursor, the suboxide tended to increase, reducing the carbide formation. The study suggested that as the percent of precursor increased in the electrospinning solution, the HER activity increased in 0.5 M H_2_SO_4_ solutions, and with 30% of metal precursor the Tafel slope, this was found to be 89 mV/dec. The interesting finding of their work was the surprisingly small value of charge transfer resistance (R_CT_), which was an order smaller than for 30% case than others, indicating a much faster kinetics for HER, resultant of increased defect-rich suboxide inclusions. 

In a separate work conducted by Tovini et al. (2018) [105], the 3D architecture of the RuO_2_/Mn_2_O_3_/carbon nanofiber (CNF) was tested as a bifunctional catalyst for the OER and ORR. The metal oxides of different morphologies were grown on electrospun CNFs separately, using the microwave synthesis route. The catalyst exhibited an onset potential of 0.95 V vs. RHE in ORR, at par with commercial Pt/C, with a Tafel slope of 121 mV/dec compared to 119 mV/dec for the latter, and an electron transfer number of 3.4, at 0.1 M KOH. For the OER, the authors have reported a value of overpotential 0.34 V at 10 mA/cm^2^. 

### 4.2. CNF Supported Metal Carbides

The nitrogen-doped carbon matrix has long been under investigation for enhanced ORR catalytic behavior. CNFs, which contain inherent nitrogen, are rich in its pyridinic N content. This N content in the carbon matrix allows more O_2_ adsorption, and thereby helps in strengthening electrode–electrolyte interactions [106]. In this sense, answers regarding N–C (nitrogen doped carbon matrix) being ORR active could be elucidated, and this helps in understanding the performance of Metal-N–C in this reaction, where metal, like Fe, acts as a promoter. A separate school of thought stresses on the fact that ORR activity is influenced by the synergistic effects of Fe–N moieties and N–C species [107]. Hu et al. (2014) [108], had demonstrated that graphite-encapsulated Fe_3_C nanoparticles, without any exposure to the electrolyte, activate the outer surface of the ORR. This has renewed the interest towards developing metal carbide, like Fe_3_C, embedded CNF architecture for fuel cell regarding studies. 

**Figure 8:**
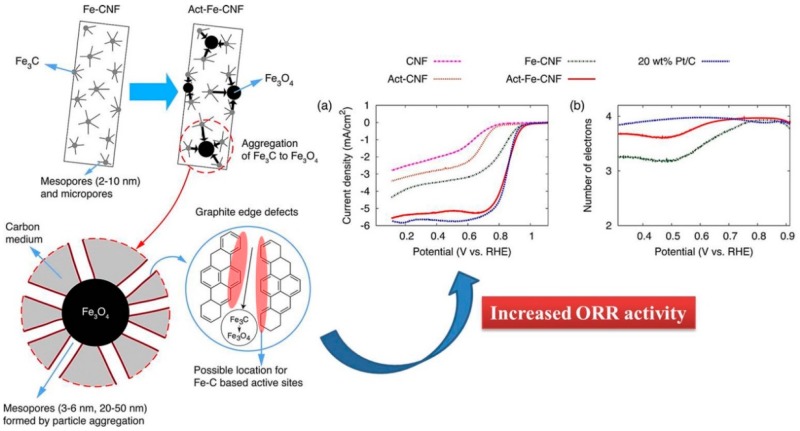
Schematic of the development of hierarchical meso/macropore structure and graphite edge defects where the potential ORR active sites are formed by water vapor activation. Image (**a**) shows the I-V curve of glassy carbon RDEs modified with metal-free CNF, vapor-activated metal-free CNF (Act–CNF), as-prepared Fe–CNF, water vapor-activated Fe–CNF for 1 h (Act–Fe–CNF), and commercial 20 wt % Pt/C, measured at a rotation rate of 1600 rpm in O_2_-saturated 0.1 M KOH (aq) solution. Image (**b**) shows electron transfer number in ORR of Act–Fe–CNF, Fe–CNF, and Pt/C. Reprinted (adapted) with permission from Ref. [107]. Copyright (2016) American Chemical Society.

**Figure 9:**
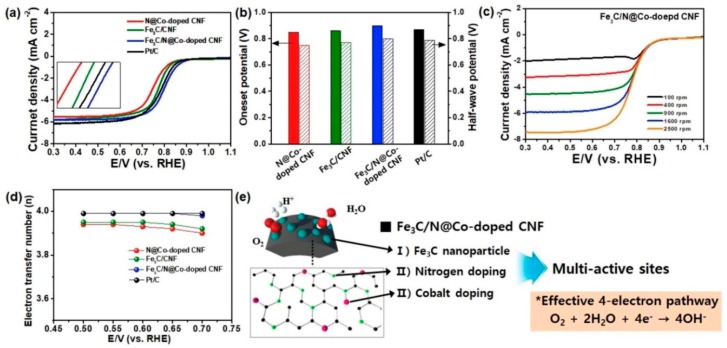
(**a**) Linear sweep voltammetry (LSV) curves of various catalysts at a rotating speed of 1600 rpm in O_2_-saturated 0.1 M KOH electrolyte. (**b**) A comparison of the onset and E_1/2_ potential from LSV curves. (**c**) LSV curves of the Fe_3_C/N@Co-doped CNF at increasing rotating speeds (**d**) Electron transfer numbers of various catalysts in a potential range of 0.5–0.7 V. (**e**) Schematic illustration of effective 4-electron pathway in Fe_3_C/N@Co-doped CNF. Reproduced with permission from Ref. [109]. Copyright Elsevier 2018.

Jeong et al. (2016) [107], had fabricated Fe_3_C–CNF via the electrospinning and carbonization technique. The prepared catalysts were further activated with water vapor, where Fe_3_C nanoparticles help in graphitization, and further activation allows F_3_C to transform into Fe_3_O_4_ which agglomerated and created a mesoporous architecture with exfoliating graphitic defect edges. As Figure 8 suggests, the activated Fe-CNF architecture is more ORR-active, and performs at a par with the commercial Pt/C, whereas the electron transfer number is also close to 4, which dictates a 4-electron pathway for the ORR, the ideal one. In a separate work conducted by An et al. (2018) [109], the authors attempted to fabricate a cost-effective, stable and efficient electrocatalyst from composite F_3_C nanoparticles on nitrogen and cobalt-doped, electrospun, carbon nanofiber (Fe_3_C/N@Co-doped CNF). The catalyst offered an onset potential of 0.9 V, with E_1/2_ of 0.8 V, and a nearly 4-electron pathway (n = 3.9) in 0.1 M KOH. The catalyst was also a better performer in chronoamperometry, and was deemed to be highly methanol-tolerant. Again, the synergistic effects of Fe_3_C and Co are well observable in Figure 9, where the complexation with nitrogen, hailing from CNF, plays a major role in ORR-improved activity. In the same area, [110] Zhao et al. (2017) demonstrated a scalable fabrication of electrospun, nitrogen-doped CNF with Fe_3_C nanoparticles-encapsulated CNTs for OER applications. The unique hierarchical architecture was rich in exposed active sites. The said catalyst exhibited outstanding performance with a high current density of 10 mA/cm^2^ at an overpotential of 284 mV, with a low Tafel slope of 56 mV/dec in a 1 M KOH solution. Alike other authors, Zhao et al. (2017) also confirmed the beauty of CNF architecture for better ion transport through the robust network. 

In the presence of basic conditions, during water splitting, Ni nanoparticles often turn into Ni(OH)_2_ which is OER active, albeit they also tend to agglomerate and corrode in a strong alkaline medium. Also, as suggested by Figure 1, Ni has weaker hydrogen binding energy (HBE) or Δ*G*_H*_. In order to mitigate this problem and to develop a catalyst which can be both HER- and OER-active [111], Li et al. (2019) coupled Ni with Mo_2_C on electrospun CNF in order to harvest the fruits of strong HBE between the latter and hydrogenm and the latter’s strong corrosion resistance. 

Although several other researchers have demonstrated the capability to contribute in OER and ORR activities, when coupled with CNTs [112], not many articles could be observed for Ni/Mo_2_C coupling on CNF for overall water splitting. Li et al. (2019) reported that N-doped Ni/Mo_2_C-based electrospun CNFs were optimized with all the necessary electrospinning parameters and Ni, Mo_2_C precursors. Ni/Mo_2_C(1:2)-NCNFs (N-doped CNFs) demonstrated the best electrocatalytic activities 1 M KOH (aq) solution, with low overpotentials of 143 mV (at the current density of 10 mA/cm^2^) for the HER and 288 mV (at current density of 10 mA/cm^2^) for the OER. The same catalyst was able to withstand 100 h of water splitting current density of 10 mA/cm^2^ at 1.64 V, when it was used as both anode and cathode. With the same concept of using Ni and Mo_2_C as a functional catalyst material strongly coupled on electrospun CNFs, Sun et al. (2019) [113] reported that the electrospinning of PVP (precursor of CNF) and NiMoO_4_ (precursor of Ni-Mo_2_C) as homologous bimetallic precursor results in in-situ interface or ridge formation between Ni and Mo_2_C phases, which opens up new active sites for HER activity, where the CNF provides the necessary structural and electrical stability. In their work, they reported the Tafel slope value as 54.7 mV/dec of the catalyst, as compared to 33.5 mV/dec of commercial Pt/C during the HER in alkaline medium.

### 4.3. CNF Supported Metal Phosphides

Transition metal phosphides are known for their excellent chemical properties due to multielectron orbitals, suitable electronic configuration and metalloid presence [114,115]. In this regard, metal phosphides like NiCoP have garnered special attention to the researchers, because they ensemble one of the most active transitional metals, Ni and Co. 

**Figure 10:**
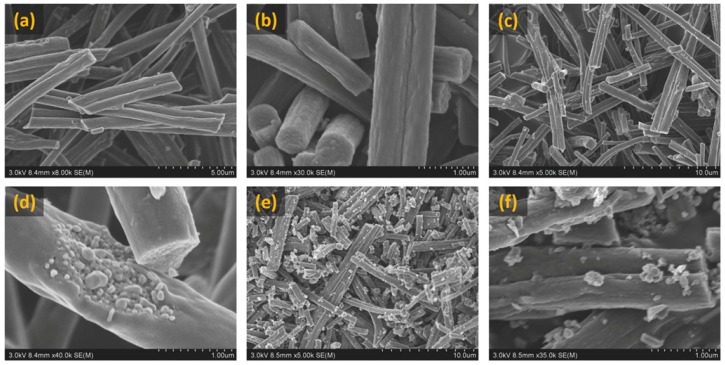
FESEM images showing NiCoP/CNF sample carbonized at 700, 800, 900 °C: (**a**,**b**) NiCoP/CNF carbonized at 700 °C, (**c**,**d**) NiCoP/CNF carbonized at 800 °C and (**e**,**f**) NiCoP/CNF carbonized at 900 °C. Reproduced with permission from Ref. [116]. Copyright John Wiley and Sons 2018.

In a recent work conducted by Surendran et al. 2018 [116], NiCoPs encapsulated in electrospun CNF were tested for energy conversion by carrying out HER, OER and ORR studies in the KOH electrolyte. As can be seen in Figure 10, NiCoP particles become more exposed to the exterior with increments in carbonization temperature. Apart from its high energy storage capability, beyond the scope of this review, the NiCoP/CNF@900 °C sample showed the best trifunctional nature to cater all the HER, OER and ORR capabilities, proving its worth for electrolyzer/metal–air batteries/fuel cell applications. 

The enhanced surface area and with conductive support provided by CNF, coupled with the bimetallic phosphide presence, ensured the high activity of the said catalyst for the ORR where the 900 °C carbonized samples exhibited E_1/2_ of 0.82 V and 7.16 mA/cm^2^ at 1600 rpm for 0.1 M KOH solution, whereas pure NiCoP had performed much poorer, 0.75 V and 2.65 mA/cm^2^, for the latter, respectively. The electron transfer number being ∼4 is due to the synergistic effect of encroaching bimetallic phosphide particles, in case of 900 °C samples. For other samples, CNF solely takes care of the electron transfer. The same catalyst shows tremendous performance in the OER and HER, where overpotential of 268 and 130 mV was needed to attain a current density of 10 mA/cm^2^, respectively. 

**Figure 11:**
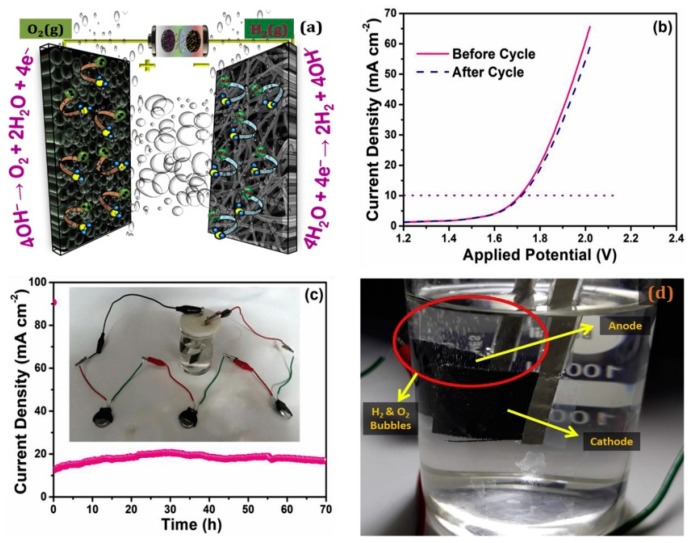
(**a**) Schematic of the lab-scale, hybrid, water electrolyzer with two asymmetric electrodes, (**b**) LSV of the device before and after a 70 h durability study, showing practically no change in its catalytic activity, (**c**) Chronoamperometry of the device for 70 h (inset) shows the supercapacitor assembly with water electrolyzer and (**d**) Photographic image of the generated O_2_ and H_2_ bubbles. Reproduced with permission from Ref. [117]. Copyright Elsevier 2019.

The same group of authors, in a separate work [117], showed the application of bimetallic catalyst containing spherically concomitant foamy NiCoP as an efficient OER and a supercapacitor electrode, and coupled that with nitrogen-doped, electrospun CNF as a superior HER-active electrode, and used the asymmetric electrode combination for the electrolyzing purpose, powering a small prototype using a supercapacitor composed of the same asymmetric electrodes (see Figure 11). The asymmetric setup required 1.71 V to produce 10 mA/cm^2^, and even after over 70 h, there was no deterioration of the electrodes. 

Wang et al. (2019) [118] synthesized N- and P-doped CNFs that were embedded with Fe_3_O_4_ and FeP, using a simple electrospinning technique that was followed by a carbonization and phosphating process. The electrochemical study of the prepared sample shows the trifunctional behavior of material towards the HER, OER and ORR, making them a suitable candidate for water splitting and fuel cells, where nitrogen-doped CNF and oxide provides the best of the OER and ORR capability, and phosphide provides the suitability towards the HER. For the ORR, its onset potential was found to be 0.017 V, 35 mV more positive than Pt/C, in 0.1 M KOH solution, and a Tafel value of 73 mv/dec was calculated. Furthermore, from the K–L analysis, the electron number was found to be 4, which makes the reactant diffusion efficient. HER studies revealed an over potential value of 149 mV at 10 mA/cm^2^ with kinetics determining the Tafel slope value of 58 mV/dec, and in case of the OER, an onset of 1.56 V and Tafel value of 90 mV/dec was obtained. From the past studies it can be easily predicted that the presence of spinel Fe_3_O_4_ and N- and P-doped carbon facilitates the electron transfer during electrochemical reaction [119,120,121,122]. 

In recent years, Gao et al. (2019) [123] fabricated a free-standing electrode containing cobalt phosphides (Co_2_P) supported via cobalt nitride moieties (CoN*_x_*) and a nitrogen, phosphorus-codoped, porous CNF (Co_2_P@CNF) via a one-step electrospinning technique. The said catalyst exhibited 0.915 V of onset potential vs. RHE (in 0.1 M KOH), comparable with 40 wt % Pt/C for ORR activities, with an electron transfer number within 3.6–3.9. For OER, Co_2_P@CNF required 42 mV additional overpotential than commercial IrO_2_ at 10 mA/cm^2^.

### 4.4. CNF-Supported Metal Sulfides

Like other transition or post-transition metal oxides, metal carbides and metal phosphides, the metal sulfides are also gaining considerable attention due to their electrical conductivity, tunable metal–sulfur coordination environment and stable chemical properties [124]. As an example, MoS_2_ possesses a high chemisorption capability for hydrogen, especially when they are under-coordinated, and thereby they show high catalytic activity for the HER. On the other hand, some researchers have also reported their OER capabilities, making such a sulfide a potential candidate for overall implementation in water splitting reactions. Zhu et al. (2015) [125], in an earlier work, had demonstrated the fabrication of a core–shell structure, comprising cubic Co_9_S_8_ as cores and layered MoS_2_ as shells on electrospun CNF. The idea was to create an interface between two catalytically active elements on a conductive, porous and tortuous network. Although the work lacked in specific comparisons between the novel catalyst and commercial ones, it indeed provides an insight to the applications of it in the electrolyzing of water. This study gave a thorough overview, both from the thermodynamic and kinetic aspects of the reactions, of the efficacy of the said catalyst. However, the major takeaway understanding of the composite was that the CNFs acted as the host for active sites for the sulfides, and protected them from erosion while giving a conductive pathway between the sulfidic core–shell nanostructures. The sulfides themselves participated in the reaction, when tested for continuous operation of 10 h at 10 mA/cm^2^ current density, and the potential requirement was −0.19 V in 0.5 M H_2_SO_4_. In a recent work conducted by Ji, et al. (2017) [126], thin MoS_2_ nanosheets grafted with MOF-derived Co–N–C flakes grown on electrospun CNFs was synthesized, aka CoNC@MoS_2_/CNF, for the HER and OER studies. The hierarchical structures with interconnected vine-like fibrous network enabled the catalyst to perform at par with commercial catalysts. 

HER studies showed a requirement of a mere 143 mV of overpotential at 10 mA/cm^2^ current with a Tafel slope of 68 mV/dec in 1 M KOH solution. In the case of OER, for the same conditions of current density, the said catalyst required 350 mV in sharp contrast with RuO_2_ (450 mV) and the Tafel slope was recorded to be 51.9 mV/dec, again superior to RuO_2_ (98.1 mV/dec). 

## 5. Conclusions

In this current review we have tried to summarize the recent trends of incorporating TM-derivative (ceramic) nanomaterials as electrocatalysts on electrospun CNF, where the latter mostly provides architectural and electrical support. Most TM derivatives have inherent electrical conductivity, which facilitates their use as electrocatalysts. On the other hand, several TM oxides suffer from poor electrical properties, specific surface area and agglomeration issues. In order to resolve the problem, and utilizing them for water splitting and fuel cell reactions, incorporating them onto electrospun CNFs are proven to be a rational concept. Electrospinning is a very simple, widely accessible, scalable and cost-effective technique, particularly suitable for the manufacturing of CNFs on an industrial scale, with a high specific surface area, enhanced porosity and tortuosity, allowing better mass transport and ionic kinetics when subjected to electrolytes. Any thermal treatment needed to modify the fiber’s physicochemical properties can be operated separately, with complete liberty to develop any hierarchical, multi-layered structure. More importantly, CNF support allows the catalysts to be distributed on a free standing binder-less electrode without using any costly electrical support like Ni-foam.

TM oxides like Co-oxides, Mn-oxides, Nb-oxides, etc., that are supported via electrospun CNFs, can overcome several of the problems mentioned above, which are otherwise encountered during energy conversion reactions, specially involving ORRs. As discussed in Section 4.1, rational designing of the catalyst material and the suitable heat treatment technique can allow the nanostructure to be altered in manner to harness specific defect sites and interfaces, like in spinel oxides, or oxygen vacancies like in the suboxides of tungsten without disturbing the structure of CNF in the electrocatalyst, which is CNF. CNFs, by their production route contain pyridinic nitrogen, which eventually renders them as heteroatom-doped carbonaceous material, and along with the oxides-defect rich structures, they prove them to be catalytically active, often at par with commercial catalysts like cost-ineffective Pt. 

In a similar path in Section 4.2, Section 4.3 and Section 4.4 other TM derivatives and their CNF composites are talked about in their various capacities as electrocatalysts for the energy conversion areas. CNFs have acted as the host material which protects the structures during polarization, whereas these other TM derivatives, because of their superior electrical properties, can outperform their oxide counterparts.

Despite the progress that has been made, some challenges still remain open: (a) Studies in alkaline conditions are well established, yet with some gray area in the mechanism but the acidic medium, especially highly acidic mediums are yet to be explored, (b) OER kinetics is still the sluggish component, and achieving a current density of 10 mA/cm^2^ for overpotential <100–150 mV should be explored further, (c) pertaining to point (b), such water splitting regarding studies should also focus on much higher specific current density to understand the most critical part of the catalyst performance, and that is stability, and finally (d) many research articles still miss to address the actual structural changes during electrochemical catalytic activity, and hence more sophisticated in-situ characterization techniques will definitely help in understanding the real problems under practical situations.
